# Patient-reported burden of intensified surveillance and surgery in high-risk individuals under pancreatic cancer surveillance

**DOI:** 10.1007/s10689-020-00171-8

**Published:** 2020-03-19

**Authors:** Kasper A. Overbeek, Djuna L. Cahen, Anne Kamps, Ingrid C. A. W. Konings, Femme Harinck, Marianne A. Kuenen, Bas Groot Koerkamp, Marc G. Besselink, Casper H. van Eijck, Anja Wagner, Margreet G. E. Ausems, Manon van der Vlugt, Paul Fockens, Frank P. Vleggaar, Jan-Werner Poley, Jeanin E. van Hooft, Eveline M. A. Bleiker, Marco J. Bruno, the Dutch Familial Pancreatic Cancer Surveillance Study Group, M. J. Bruno, D. L. Cahen, J. W. Poley, F. Harinck, I. C. A. W. Konings, K. A. Overbeek, I. J. M. Levink, B. Koopmann, A. Wagner, B. Groot Koerkamp, K. Biermann, M. P. Peppelenbosch, P. Fockens, J. E. van Hooft, M. van der Vlugt, B. A. J. Bastiaansen, M. G. Besselink, M. G. E. Ausems, M. E. Velthuizen, F. P. Vleggaar, H. van Dullemen, E. M. A. Bleiker, M. A. Kuenen

**Affiliations:** 1 Department of Gastroenterology & Hepatology, Erasmus University Medical Center, Rotterdam, The Netherlands; 2 Division of Psychosocial Research and Epidemiology, The Netherlands Cancer Institute, Amsterdam, The Netherlands; 3 Department of Surgery, Erasmus University Medical Center, Rotterdam, The Netherlands; 4 Department of Surgery, Cancer Center Amsterdam, Amsterdam UMC, University of Amsterdam, Amsterdam, The Netherlands; 5 Department of Clinical Genetics, Erasmus University Medical Center, Rotterdam, The Netherlands; 6 Division Laboratories, Pharmacy and Biomedical Genetics, Department of Genetics, University Medical Center Utrecht, Utrecht, The Netherlands; 7 Department of Gastroenterology & Hepatology, Amsterdam Gastroenterology and Metabolism, Amsterdam UMC, University of Amsterdam, Amsterdam, The Netherlands; 8 Department of Gastroenterology & Hepatology, University Medical Center Utrecht, Utrecht, The Netherlands

**Keywords:** Psychology, Patient-reported outcome measures, Resection, Quality of life, Pancreatic cancer, Surveillance

## Abstract

In high-risk individuals participating in a pancreatic cancer surveillance program, worrisome features warrant for intensified surveillance or, occasionally, surgery. Our objectives were to determine the patient-reported burden of intensified surveillance and/or surgery, and to assess post-operative quality of life and opinion of surgery. Participants in our pancreatic cancer surveillance program completed questionnaires including the Cancer Worry Scale (CWS) and the Hospital Anxiety and Depression Scale (HADS). For individuals who underwent intensified surveillance, questionnaires before, during, and ≥ 3 weeks after were analyzed. In addition, subjects who underwent intensified surveillance in the past 3 years or underwent surgery at any time, were invited for an interview, that included the Short-Form 12 (SF-12). A total of 31 high-risk individuals were studied. During the intensified surveillance period, median CWS scores were higher (14, IQR 7), as compared to before (12, IQR 9, *P* = 0.007) and after (11, IQR 7, *P* = 0.014), but eventually returned back to baseline (*P* = 0.823). Median HADS scores were low: 5 (IQR 6) for anxiety and 3 (IQR 5) for depression, and they were unaffected by the intensified surveillance period. Of the 10 operated patients, 1 (10%) developed diabetes and 7 (70%) pancreatic exocrine insufficiency. The interviews yielded median quality-of-life scores comparable to the general population. Also, after surgery, patients’ attitudes towards surveillance were unchanged (5/10, 50%) or became more positive (4/10, 40%). Although patients were aware of the (sometimes benign) pathological outcome, when asked if surgery had been justified, only 20% (2/10) disagreed, and all would again have chosen to undergo surgery. In conclusion, in individuals at high risk for pancreatic cancer, intensified surveillance temporarily increased cancer worries, without affecting general anxiety or depression. Although pancreatic surgery led to substantial co-morbidity, quality of life was similar to the general population, and surgery did not negatively affect the attitude towards surveillance.

**Electronic supplementary material:**

The online version of this article (10.1007/s10689-020-00171-8) contains supplementary material, which is available to authorized users.

## Introduction

Pancreatic cancer surveillance aims to detect pancreatic ductal adenocarcinoma (PDAC) or its precursors in a resectable stage. Because a non-invasive and accurate diagnostic test is lacking, and the incidence of PDAC in the general population is low [1], surveillance is only recommended for selected individuals with an increased lifetime risk [2]. For this group, magnetic resonance imaging (MRI) and/or endoscopic ultrasonography (EUS) at twelve-month intervals is advised [2]. The goal is to detect stage one PDAC or, preferably, high-grade precursor lesions [2]. However, especially in small lesions, imaging techniques may not be able to distinguish malignancy and high-grade precursors from benign disease [3, 4].

As a consequence, in large prospective surveillance programs, up to 50% of patients undergo surgery for low-grade or non-dysplastic lesions [5–7]. And despite recent advances, pancreatic surgery is still associated with substantial morbidity and mortality [8]. Cases have been reported of high-risk individuals experiencing nearly fatal complications after resection of a lesion that did not harbor malignancy or high-grade dysplasia [9]. It may be argued that surgery was unjustified in these cases, as the goals of surveillance were not met. On the other hand, we do not know the progression rate of pancreatic lesions in high-risk individuals, and what the outcomes would have been, had surgery been postponed. It is unknown how high-risk individuals perceive this dilemma and whether they find the burdens of surgery to outweigh the gains.

To prevent surgery for benign disease, individuals with lesions of unknown relevance are usually subjected to an intensified surveillance period, with shortened intervals of three or six months [2]. During this time, patients are burdened with the knowledge of having a lesion of unclear etiology, and with undergoing additional visits and investigations. Reinforced by the oftentimes strong family history of PDAC, this seems an obvious cause for worries. While earlier studies have shown that, on average, the psychological burden of surveillance seems acceptable [10], the psychological impact of a period of intensified surveillance has never been assessed.

Therefore, the aims of this study were to assess: (1) the patient-reported impact of intensified surveillance on cancer worries, anxiety, and depression; and (2) the patient-reported burden of surgery, and the post-operative quality of life and opinion of surgery.

## Methods

### Design of the surveillance study

This study is part of the ongoing Dutch familial pancreatic cancer surveillance study (FPC-study). This prospective study, started in 2006, is performed in three university hospitals and investigates the effectiveness of pancreatic cancer surveillance in high-risk individuals. Eligible for surveillance are asymptomatic individuals with an estimated 10% or greater lifetime risk of PDAC, encompassing carriers of a mutation in a known pancreatic cancer susceptibility gene and individuals without a known gene mutation but a strong family history of PDAC, defined as familial pancreatic cancer (FPC) kindreds. Complete inclusion criteria are listed in Box 1. The minimal age for inclusion was 45 years until 2013 and 50 thereafter, or ten years younger than the age of the youngest relative with PDAC, whichever of the two ages was the lowest. Surveillance was stopped at the age of 75. A clinical geneticist evaluated all individuals prior to enrollment. If a mutation in a known PDAC susceptibility gene was found (see Box 1), only family members who tested positive were enrolled.

**Box 1:** Inclusion criteria for the Dutch Familial Pancreatic Cancer Surveillance Study (FPC-study). Harinck et al. [11]

1. *CDKN2A* gene mutation, regardless of PDAC family history	
2. Peutz-Jeghers syndrome (proven *LKB1/STK11* gene mutation or clinical diagnosis), regardless of PDAC family history	
3.* BRCA2*,* BRCA1*,* TP53*,* MLH1*,* MSH2* or* MSH6* gene mutation, and ≥ 2 relatives with PDAC, of which ≥ 1 histologically proven	
4. First-degree relatives of a family member with PDAC, in families with ≥ 1 histologically proven PDAC, and either:	
(a) PDAC in ≥ 2 relatives who were first-degree relatives to each other	
(b) PDAC in ≥ 3 relatives, who were first or second-degree relatives to each other	
(c) PDAC in ≥ 2 relatives, of which ≥ 1 was under 50 years of age, who were first or second-degree relatives to each other	

*BRCA* Breast Cancer, *CDKN2A* Cyclin-Dependant Kinase Inhibitor 2A, *LKB1/STK11* Liver Kinase B1/Serine/Threonine Kinase 11, *MLH* MutL Homolog, *MSH* MutS Homolog, *PDAC* Pancreatic Ductal Adenocarcinoma, *TP53* Tumor Protein 53

### Surveillance procedures and clinical management

The study procedures have been described previously [11–13]. In summary, at baseline and follow-up visits, both EUS and MRI were performed. Since 2009, participants have been invited to complete psychological questionnaires following each surveillance visit [12, 14, 15]. Clinical management was decided upon by a multidisciplinary expert panel, consisting of endosonographists, radiologists, surgeons, and pathologists. The policy was as follows:

1. Regular surveillance after twelve months in case of no abnormalities, minor signs of chronic pancreatitis, or cystic lesions without worrisome features.
2. Intensified surveillance after three or six months when a worrisome lesion was detected not warranting immediate surgery. This included indeterminate solid lesions; cystic lesions with a worrisome feature but no high-risk stigmata (e.g. a thickened enhanced cyst wall, cyst growth of 5 mm/2 years, or mural nodule < 5 mm) [16]; and a dilated main pancreatic duct of < 10 mm without a visible mass. If a lesion remained stable in size and/or was no more considered suspicious for malignancy, the surveillance interval was reversed to twelve months.
3. Surgical resection was performed if the expert panel agreed on suspicion for malignancy, based on positive cytology; a main pancreatic duct dilation ≥ 10 mm and/or an abrupt caliber change; a cystic lesion with high-risk stigmata or ≥ two worrisome features [16]; or a solid lesion.

### Patient selection

**Fig. 1:**
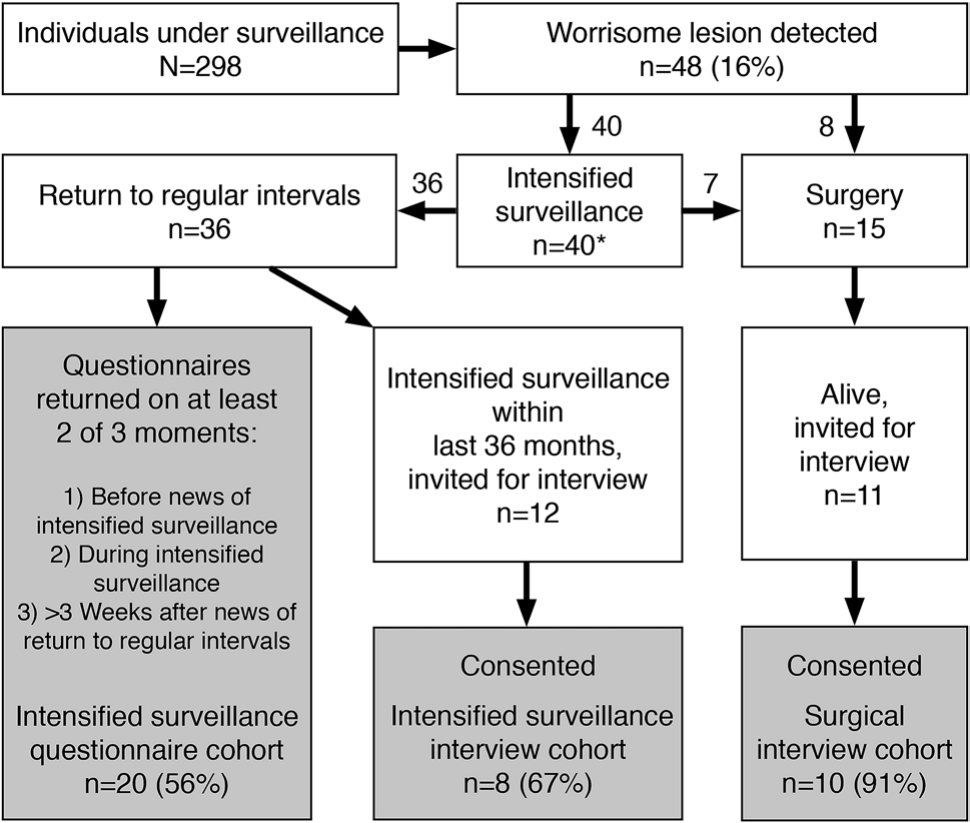
Flow-chart of patient selection and response rate per studied subcohort. Note: *Three patients underwent two separate intensified surveillance periods, separated by several years, leading to a return to regular intervals in one period, and to surgery in the other

A flow chart of the patient selection process is shown in Fig. 1. We identified all individuals in whom a worrisome lesion had been detected for which they had undergone intensified surveillance and/or surgical resection by January 2018. These patients were classified into three subcohorts:

1. The intensified surveillance questionnaire subcohort consisted of those who had returned to regular intervals and who had completed at least two out of the three questionnaires: (1) while under regular surveillance, before the decision to intensify surveillance; (2) during the intensified surveillance period; and (3) ≥ 3 weeks after the decision to return to regular intervals.
2. The intensified surveillance interview subcohort consisted of those who had returned to regular intervals and consented to an interview. To reduce recall bias, this was restricted to those who underwent intensified surveillance within the last three years. Individuals could be included in both the questionnaire and the interview subcohort.
3. The surgical interview subcohort consisted of those who underwent surgical resection (with or without prior intensified surveillance period) and consented to an interview. For these interviews, inclusion was not restricted and all who underwent surgery and were alive were invited.

### Psychological questionnaires

We investigated the questionnaire outcomes regarding cancer-related worries, general anxiety, and depression. These were measured using the Cancer Worry Scale (CWS) and the Hospital Anxiety and Depression Scale (HADS). The CWS is a validated tool containing eight items, of which the total score ranges from eight to 32. A score of ≥ 14 is indicative of high levels of cancer worries [17, 18]. The HADS is a validated tool consisting of two subscales: one for general anxiety (HADS-A) and one for depression (HADS-D). Each contains seven items and scores for each range from zero to 21 [19–21]. A score of ≥ 8 is considered to reflect elevated levels of general anxiety and depression [21].

### Semi-structured interviews

Patients were interviewed by telephone by the same medical doctor (AK). The interviews were semi-structured, meaning there was a structure of topics that needed to be covered using pre-defined, open-ended questions. If deemed appropriate, the interviewer could deviate from the structure in order to follow certain topics [22]. The interviews included questions on their general opinion of surveillance, and specifically on either the intensified surveillance period or surgery (complete interview structures in the Supplementary Information). The interview for surgical patients also included the Short Form-12 (SF-12), a validated questionnaire to measure physical and mental quality of life [23, 24]. This score ranges from zero to 100, with higher scores indicating better quality of life, and a score of 50 representing the mean in the general population [24, 25].

The audiotaped interviews were transcribed and analyzed using conventional content analysis [26]. Two independent reviewers (KAO and AK) interpreted and labelled the responses using a predefined codebook. The codebook contained response categories that were drawn up based on the expected range of answers, both positive and negative. Both reviewers labelled the first interview of both the intensified surveillance interview and the surgery interview, and then discussed discrepancies to reach consensus on how to label responses. After labelling the first half of the interviews, another interview of each type was labelled by each reviewer and then discussed before the remainder was labelled.

### Statistical analysis

Descriptive statistics are presented as percentages, or as medians with interquartile range or means with standard deviation, depending on the distribution of the data. Cohen’s kappa was performed on the interview results to assess inter-rater agreement. The Cronbach’s alpha on internal consistency was performed on the CWS, the HADS-A and HADS-D to assure reliability. All three showed good internal consistency (α > 0.8) at each of the three time points (before, during and after intensified surveillance). Changes in CWS, HADS-A and HADS-D scores could not be analyzed with a linear mixed model, because the underlying assumption that missing questionnaires were missing at random could not be made. Instead, these changes were assessed using the Wilcoxon signed rank test, which was performed on the three different combinations of repeated measurements (before versus during intensified surveillance, during versus after, and before versus after). We corrected for this multiple testing of each scale by applying a Bonferroni correction (0.05/3). A *P* value of < 0.017 was considered statistically significant. To compute the physical and mental component summaries (PCS and MCS) of the SF-12, regression weights were used that were derived from normative data of the Dutch general population, using the orthogonal rotation method [24]. We used SPSS Statistics 22 (IBM Corporation, Armonk, New York, USA) for all analyses.

## Results

### Response rates and patient characteristics

By January 2018, 298 individuals were under pancreatic cancer surveillance, of whom 36 (12%) had been subjected to a period of intensified surveillance not leading to surgery or a diagnosis of PDAC (Fig. 1). In five individuals, this took place prior to commencement of the questionnaire study in 2009. Of the remaining 31, 20 (65%) had completed questionnaires on at least two of the three occasions (before, during, and after intensified surveillance), and were included in the questionnaire subcohort. Twelve individuals underwent intensified surveillance within the last three years and were invited for an interview. Two (17%) did not respond, two (17%) declined participation without explanation, and eight individuals (67%) consented. Five of the eight were also included in the questionnaire subcohort. Therefore, in total, 23 out of 31 (74%) eligible intensified surveillance participants were studied.

Of the 15 patients who underwent surgery, four died of PDAC. Of the 11 living individuals, one declined participation without explanation and ten (91%) consented to an interview. Two of these patients were also included in the questionnaire subcohort (for an intensified surveillance period that was at least a year apart and independent from their surgery). Altogether, 31 individuals were studied: 12 (39%) male; median age 52 (IQR 13) years; 16 (52%) familial pancreatic cancer kindreds; and 15 (48%) gene mutation carriers. Baseline characteristics of the three subcohorts are presented in Table 1.

**Table 1:** Characteristics of intensified surveillance patients and surgical patients

Patient characteristics	Intensified surveillance		Surgery
	Questionnaires (n = 20)	Interviewees (n = 8)	Interviewees (n = 10)
Age at start surveillance, median (IQR), y	51 (11)	53 (15)	46 (11)
Time under surveillance, median (IQR), m	99 (40)	68 (81)	88 (72)
Male gender	7 (35)	4 (50)	5 (50)
Caucasian	18 (90)	8 (100)	9 (90)
**Risk category**			
Familial Pancreatic Cancer kindred	8 (40)	5 (63)	7 (70)
Mutation carrier	12 (60)	3 (38)	3 (10)
* CDKN2A* (FAMMM syndrome)	7 (35)	2 (25)	1 (10)
* BRCA2* + 2 affected family members (HBOC)	3 (15)	1 (13)	1 (10)
* STK11/LKB1* (Peutz-Jeghers syndrome)	1 (5)	0 (0)	1 (10)
* TP53* (Li Fraumeni syndrome)	1 (5)	0 (0)	0 (0)
**Number of relatives with PDAC**			
0	2 (10)	1 (13)	1 (10)
1 or 2	10 (50)	3 (38)	3 (30)
3 or more	8 (40)	4 (50)	6 (60)
Age youngest relative with PDAC, median (IQR)	56 (16)	61 (29)	51 (11)
Personal history of non-pancreatic malignancy	12 (60)	5 (63)	3 (30)
**Education level**			
Low (only primary or secondary school)	5 (25)	2 (25)	3 (30)
Medium (education after secondary school)	5 (25)	3 (38)	4 (40)
High (college or university)	10 (50)	3 (38)	3 (30)
**Has children**			
Yes	17 (85)	6 (75)	6 (60)
No	2 (10)	0 (0)	1 (10)
Unknown	1 (5)	2 (25)	3 (30)
**Married or co-habiting**			
Yes	13 (65)	4 (50)	7 (70)
No	6 (30)	2 (25)	0 (0)
Unknown	1 (5)	2 (25)	3 (30)

Values presented as n (%) unless otherwise indicated

*BRCA,* Breast Cancer, *CDKN2A,* Cyclin-Dependant Kinase Inhibitor 2A, *FAMMM* familial atypical multiple mole melanoma,* HBOC* hereditary breast and ovarian cancer,* IQR* interquartile range, *LKB1/STK11* Liver Kinase B1/Serine/Threonine Kinase 11, *PDAC* pancreatic ductal adenocarcinoma, *TP53* Tumor Protein 53

### Cancer worries, Anxiety, and Depression

**Fig. 2:**
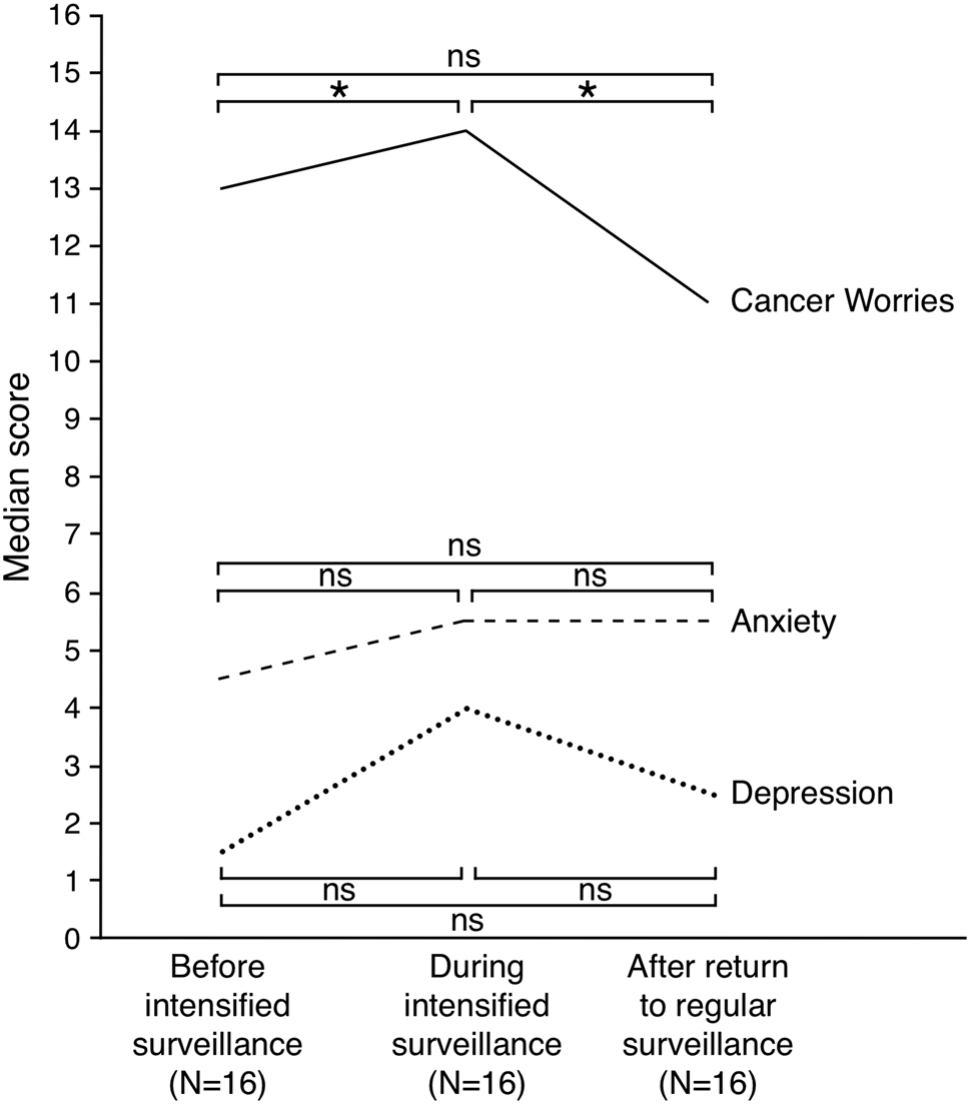
Median Cancer Worry Scale (CWS) and Hospital Anxiety and Depression Scale (HADS) scores before, during, and ≥ 3 weeks after an intensified surveillance period. Note: Graph displays median scores of all patients per time point, statistical analyses were performed on paired measurements only (before versus during, during versus after, before versus after, n = 12 for all three analyses). Significant differences marked with *, non-statistically significant differences with ns

Median scores are visualized in Fig. 2. Cancer worries were significantly higher during intensified surveillance (median 14, IQR 7) than before (median 12, IQR 9, *P* = 0.007), and decreased significantly after (median 11, IQR 7, *P* = 0.014). After surveillance resumed at regular intervals, cancer worries returned to baseline levels (*P* = 0.823). Overall anxiety (median 5, IQR 6) and depression (median 3, IQR 5) scores were low and did not change during or after intensified surveillance (all *P* values > 0.017).

### General opinion on surveillance

The results of the 18 interviews on surveillance are presented in Table 2. Interviewees reported their motivation to participate in surveillance to be: achieving early detection of pancreatic cancer (15/18, 83%), contributing to research (9/18, 50%), improving prognosis in the future for themselves and/or younger family members (4/18, 22%), and making them feel in control or proactive (3/18, 17%). Their preferred surveillance interval was twelve months (8/18, 44%), twelve months but shorter if abnormalities are detected, in concordance with the current protocol (4/18, 22%), or six months (4/18, 22%). One patient preferred to leave it up to the specialist’s opinion and another to decide after discussion with the specialist. The vast majority (17/18, 94%) wanted long-term surveillance, either forever (8/18, 44%) or until it is stopped by the program (9/18, 50%). Regarding surveillance modalities, EUS was preferred over MRI by 50% (9/18), MRI over EUS by 11% (2/18), and 28% (5/18) did not have a preference.

**Table 2:** Interview results of general questions on surveillance

	Intensified surveillance	Surgery	Total
	n = 8	n = 10	N = 18
**Motivation to participate (could be more than one)**			
To detect pancreatic cancer early	8 (100)	7 (70)	15 (83)
To help scientific research	6 (75)	3 (30)	9 (50)
To improve prognosis	1 (13)	3 (30)	4 (22)
To have the feeling to have influence/control	0 (0)	3 (30)	3 (17)
**Preferred surveillance interval**			
12 months	3 (38)	5 (50)	8 (44)
12 months but shorter if abnormalities present	1 (13)	3 (30)	4 (22)
6 months	2 (25)	2 (20)	4 (22)
Decide together with doctor	2 (25)	0 (0)	2 (11)
**Preferred surveillance duration**			
Forever	4 (50)	4 (40)	8 (44)
Until a certain age	1 (13)	0 (0)	1 (6)
Until no longer required by the surveillance program	3 (38)	6 (60)	9 (50)
**Preferred imaging modality**			
EUS	4 (50)	5 (50)	9 (50)
MRI	2 (25)	0 (0)	2 (11)
No preference	2 (25)	3 (30)	5 (28)
Missing	0 (0)	2 (20)	2 (11)

Values presented as n (%)

*EUS* endoscopic ultrasonography, *MRI* magnetic resonance imaging

### Intensified surveillance interviews

The inter-rater agreement of the first interview was good (κ 0.695, *P* < 0.001) and improved to outstanding halfway (κ 0.835, *P* < 0.001). Intensified visits were experienced as conveying more anxiety or nervousness by three individuals (38%), as more burdensome by two (25%), and as no different from regular follow-up by two (25%). One patient reported not to have experienced more anxiety, but even to be grateful to have undergone additional imaging that provided swift reassurance. According to the participants, the regular follow-up schedule was resumed because the lesion remained stable in size (four individuals, 50%), because the lesion was determined to be benign (one, 13%), and for reasons unknown to the participant (three individuals, 38%). Five (63%) individuals still worried over the detected abnormality, despite having returned to regular intervals. Two patients judged the intensified surveillance period as something positive (25%), two as neutral (25%), three as negative but necessary (38%), and one as just negative (13%). The majority (75%) of patients claimed that their general opinion of surveillance did not change by undergoing a period of intensified surveillance. One (13%) now thought more positively towards surveillance and one more negatively. Three individuals (38%) actively worried about their family members, and none would discourage family members to undergo surveillance.

### Surgery interviews

The ten interviewed surgical patients were operated at a mean age of 51 (SD 8.6) years. They were interviewed a median of 43 (IQR 63) months after surgery. Again, the inter-rater agreement of the surgery interview was good at first (κ 0.725, *P* < 0.001) and outstanding halfway (κ 0.831, *P* < 0.001). The complete results of the interviews are listed in Table 3.

**Table 3:** Interview results of surgery questions

	Age at surgery/time since surgery	Surgery type	Recovery^a^	Current health^b^	DM	Pancreatic exocrine deficiency	PA outcome (report)^c^	PA outcome (patient)	Surgery justified (patient’s opinion)	Opinion of surgery	Would again have chosen surgery	Opinion of surveillance changed	Worries for family	Would recommend surveillance to family
1	56y/108 m	PD	Fair	Fair	N	Y	LGD	Benign	N	Neutral	Y	Unchanged	N	Y
2	46y/95 m	PD	Poor	Fair	N	Y	LGD	Precursor	Y	Negative	Y	Unchanged	N	Missing
3	47y/72 m	DP	Fair	Missing	Y	Y	LGD	Precursor	Y	Negative	Y	Positively	Y	Y
4	49y/56 m	DP	Fair	Good	N	N	LGD	Precursor	Y	Necessary	Y	Positively	Y	Y
5	47y/56 m	DP	Fair	Good	N	N	LGD	Precursor	Y	Positive	Y	Unchanged	Missing	N/A
6	57y/29 m	PD	Poor	Good	N	Y	NET	Malignant	Y	Negative	Y	Unchanged	N	Missing
7	32y/24 m	DP	Good	Fair	N	Y	AIP	Benign	N	Necessary	Likely	Unchanged	Y	Y
8	49y/18 m	DP	Good	Good	N	N	LGD	Benign	Y	Positive	Y	Positively	N	Y
9	64y/6 m	PD	Poor	Fair	N	Y	MGD	Precursor	Y	Necessary	Y	Positively	N	N
10	54y/4 m	PD	Fair	Fair	N	Y	PDAC	Unknown	Unknown	Positive	Y	Missing	N	N/A

^a^Recovery as rated by patient: Good if fast and without complications, Fair if minor complications and/or longer recovery time than anticipated, Poor if major complications

^b^Health as rated by patient: Good if the same as before surgery, Fair if improving but not quite the same as before surgery

^c^In case of multiple lesions in the resected specimen the highest grade of dysplasia was stated. LGD, pancreatic intraepithelial neoplasia 1 or 2, side branch intraductal papillary mucinous neoplasm with low or moderate-grade dysplasia; MGD, mixed-type intraductal papillary mucinous neoplasm with low-grade dysplasia; NET, neuroendocrine tumor; AIP, auto-immune pancreatitis; PDAC, pancreatic ductal adenocarcinoma

*DM* diabetes mellitus, *PA* pathology, *y* years, *m* months, *PD* pancreatoduodenectomy, *DP* distal pancreatectomy, *Y* yes, *N* no, *N/A* not applicable (no family members or are not eligible for surveillance)

Of the surgical patients, two individuals (20%) judged their immediate recovery after surgery as good (fast, no complications), five individuals (50%) as fair (longer recovery time than anticipated and/or minor complications), and three individuals (30%) as poor (major complications). At the time of interview, four (40%) had returned to their preoperative health level (median 43 months after surgery, IQR 35) and five individuals (50%) had not completely recovered (median 24 months after surgery, IQR 97). One patient had developed diabetes mellitus requiring insulin therapy, and seven (70%) had developed exocrine pancreatic insufficiency, leading to digestive complaints and changes in fecal consistency requiring dietary changes and enzyme replacement therapy at every meal.

Patients’ interpretation of the pathological outcome showed moderate agreement (κ 0.500, *P* = 0.007) with the actual pathology report. The one person with pancreatic cancer (more specifically, intraductal papillary mucinous neoplasia-associated invasive carcinoma), was unaware of this outcome, despite detailed explanation while admitted to the hospital post-surgery. The majority (seven individuals, 70%) deemed surgery to have been justified. The two who did not, had benign pathological outcomes. When asked if they would choose surgery again—if presented with the same situation—all patients affirmed, including the three who believed they had undergone surgery for benign disease, the three who judged surgery as something negative, and the patient who was unaware of the malignant pathological outcome.

Their general opinion of surveillance was unchanged by surgery (five individuals, 50%) or positively affected (four individuals, 40%), but never negatively. Only one person (who had developed major complications, including a hepaticojejunostomy stenosis with recurrent cholangitis, and abdominal abscesses requiring several re-admissions) would not actively recommend surveillance to family members, but would leave the choice up to them. All other patients would recommend surveillance.

### Quality of life after surgery

Median quality-of-life scores (median 43 months after surgery, IQR 6) were 56 (IQR 5) for the physical component summary, and 52 (IQR 7) for the mental component summary. These scores were comparable to age-matched normative data from the Dutch general population [24].

## Discussion

This is the first study that specifically investigates the burden of intensified surveillance and surgery, as perceived by high-risk individuals participating in a pancreatic cancer surveillance program. It addresses several potential harms of surveillance, including false positive test results; complications and side effects of diagnostic investigations and treatment; overtreatment; and the associated psychological burden [27]. Assessment and avoidance of harm is of great importance in cancer screening [28]. This holds especially true for pancreatic cancer surveillance, as benefits in reducing cancer-related mortality are still unproven, and treatment is associated with significant morbidity or even mortality [8]. Additionally, indeterminate lesions are frequently found (in our cohort in 16%), making assessment of the burden of intensified surveillance highly relevant.

Previously, results from our and other groups demonstrated, on average, low cancer worries, general anxiety, depression and general distress within surveillance cohorts of high-risk individuals [14, 15, 29–32]. This was recently confirmed in a systematic review [10]. Earlier, we described an increase in CWS score of six individuals under intensified surveillance, but this did not reach statistical significance [12].

Presently, we were able to analyze a larger number of individuals and demonstrated that intensified surveillance leads to a temporary increase of cancer worries reaching the cut-off value for a high level of fear. Indeed, it is understandable that finding an indeterminate lesion and undergoing additional investigations lead to an increase in worries in individuals with high PDAC risk. It is reassuring that this increase is relatively small (median of two on a scale from 8 to 32), and temporary. In the interviews, five out of eight individuals reported to still regularly worry about the abnormality that was found, despite having returned to regular intervals. However, their CWS scores at that time point were relatively low, suggesting these worries are also moderate and do not interfere with their daily functioning. Overall, the burden of intensified surveillance seems tolerable, which renders the decision to closely monitor an indeterminate lesion more justified and much preferred over surgery, with its associated risks and the possibility of overtreatment. This is especially true given the result that of 48 individuals with a worrisome lesion, only two were diagnosed with a malignancy or pathologically proven high-grade dysplasia.

After surgery, the pathological outcomes of the majority (80%) of individuals did not fulfill formal surveillance goals (seven resected with only low-grade dysplasia, one non-required resection for autoimmune pancreatitis). There is still no consensus amongst experts whether these cases should be considered a success of surveillance or as overtreatment [2]. Nevertheless, most patients deemed surgery to have been justified and all of them would likely or definitely choose to undergo surgery again. This may be a reflection of high-risk individuals being highly motivated to participate in surveillance and undergo treatment, driven by a strong will to prevent dying of pancreatic cancer. It may also have been influenced by cognitive dissonance. Cognitive dissonance is a psychological discomfort or tension that can arise when there is a discrepancy between an individual’s actions and attitude [33]. This can occur especially in situations in which the action is irreversible and was the individual’s own choice, and when there are negative outcomes. In order to reduce this psychological discomfort, individuals may adjust their attitudes to support their initial actions by increasing their preference for the selected option [34]. In addition, participants’ positive opinion of surgery may be attributable to the participants being insufficiently aware of the pathological outcome, given the moderate agreement between the diagnosis according to the pathologist and the patient.

Another finding of insufficient awareness was the fact that multiple patients were unprepared for the physical invalidation and long recovery time after surgery. It has been recognized that information on cancer screening rarely provides details about potential negative outcomes [28]. Our results show that also in our program there is room for improvement in ensuring patients’ understanding of the surgical procedure, the recovery process, and the pathological outcome. This is essential to help individuals make an informed decision regarding whether they opt for undergoing surveillance and surgery [27].

At the time of interviewing, despite the development of pancreatic exocrine insufficiency in the majority, all operated patients judged their health as “fair” or “good”, and quality of life scores were similar as age-matched reference data from the general population [24]. Post-surgery quality of life has not previously been reported for high-risk individuals in a pancreatic cancer surveillance program. Most studies have been performed in either pancreatic cancer or chronic pancreatitis patients, whose prospects and physical state are incomparable to our cohort. Other studies have reported on a more similar group, consisting of patients undergoing surgery for benign pancreatic disease other than chronic pancreatitis, and found similar results as ours, showing good quality-of-life scores that were not negatively impacted by surgery [35–38].

Several strengths and limitations of our study can be noted. The reliability of our results is assured by several methodical strengths. These include a prospective design and the use of validated tools to measure psychological burden, which all showed good internal consistency. Furthermore, the semi-structured interviews yielded high response rates. In addition, there was a good-to-outstanding inter-rater agreement in the interpretation of the interviews, although the number of double-labelled interviews was relatively low and hence, this could partly be due to chance.

Although our surveillance program in individuals at high risk for pancreatic cancer is one of the largest in the world, the number of participants who had undergone intensified surveillance and/or surgery was limited, and not all eligible individuals had returned questionnaires at all three time points. A larger eligible population would have made our results more robust and could have shown differences between groups that currently did not reach statistical significance. Because the used interval during intensified surveillance is often three months, this leaves a small window for the questionnaire to be sent out and returned. In order to keep the results valid, we excluded questionnaires with unreliable timing. We have overcome this limitation by including all participants who returned a questionnaire at two of the three time points, and by performing the additional interviews. Ultimately, we were able to assess the impact of intensified surveillance in 74% of the eligible individuals. It has to be noted that, as we do not have data on the individuals who declined participation in the surveillance program, we should only extrapolate our results to individuals who undergo surveillance. Secondly, no specific validated questionnaire was available for our surgical patients. Although there are specific validated tools for patients operated for cancer, these do not apply to our cohort of individuals who are at risk, but do not have a cancer diagnosis. We used the SF-12, a more general validated tool to measure quality of life, with available normative data from the Dutch general population [23, 24]. A third limitation is the possible effect of a recall bias. As with all retrospective interviews, participants’ may be biased in their memories of an event several years prior, and their opinion may be more moderate than at the time of the event. To limit this, we restricted inclusion to individuals who underwent an intensified surveillance period a maximum of three years prior. Of the final eight participants, only two (25%) underwent intensified surveillance more than two years prior. Regarding the surgical interviews, all surgical patients were invited, without restriction on the date of surgery. Because pancreatic surgery can lead to permanent long-term co-morbidities (such as pancreatic exocrine insufficiency and insulin-dependent diabetes mellitus) which often require time to develop, it is not preferred to assess the impact of surgery shortly after the event.

In summary, the burden of intensified investigations within a surveillance program for individuals at high risk for pancreatic cancer seems tolerable. Overall, it did not influence the positive attitude of high-risk individuals towards the pancreatic cancer surveillance program, and the increase in cancer worries was transient and relatively modest. Our results suggest the burden of surgery is acceptable to high-risk individuals undergoing pancreatic cancer surveillance, and perceived to be outweighed by the benefit of possible early detection and curation of pancreatic cancer. However, taking into account the associated morbidity and mortality of pancreatic surgery, the difficulty of correctly identifying high-risk lesions based on imaging, and that the majority of these so-called indeterminate lesions in our cohort eventually concerned irrelevant lesions, the decision to perform surgery should be carefully made after discussion within a multidisciplinary panel.

## Electronic supplementary material

Below is the link to the electronic supplementary material.

Electronic supplementary material 1 (PDF 114 kb)
