# Supplementary material for: Patient-reported burden of intensified surveillance and surgery in high-risk individuals under pancreatic cancer surveillance
Source: Fam Cancer. 2020 Mar 19;19(3):247–58. doi: 10.1007/s10689-020-00171-8 (PMC7242488; doi:10.1007/s10689-020-00171-8)
Supplement: Supplementary file 1 — Electronic supplementary material 1 (PDF 114 kb) [file 10689_2020_171_MOESM1_ESM.pdf]

Supplementary Information

Patient-reported burden of intensified surveillance and surgery in high-risk individuals under pancreatic cancer surveillance

JOURNAL

Familial Cancer

AUTHORS

Kasper A. Overbeek, MD, Djuna L. Cahen, MD, PhD, assistant professor, Anne Kamps, MD, Ingrid C.A.W. Konings, MD, PhD, Femme Harinck, MD, PhD, Marianne A. Kuenen, Bas Groot Koerkamp, MD, PhD, Marc G. Besselink, MD, PhD, professor, Casper H. van Eijck, MD, PhD, professor, Anja Wagner, MD, PhD, Margreet G.E. Ausems, MD, PhD, associate professor, Manon van der Vlugt, MD, PhD, Paul Fockens, MD, PhD, professor, Frank P. Vleggaar, MD, PhD, professor, Jan-Werner Poley, MD, PhD, Jeanin E. van Hooft, MD, PhD, MBA, associate professor, Eveline M.A. Bleiker, PhD, professor, Marco J. Bruno, MD, PhD, professor. On behalf of the Dutch Familial Pancreatic Cancer Surveillance study group.

CORRESPONDING AUTHOR

Kasper A. Overbeek
Department of Gastroenterology & Hepatology
Erasmus University Medical Center, Rotterdam, The Netherlands
E-mail address: k.overbeek@erasmusmc.nl
Telephone: 0031 (0)6 13 69 32 76
Fax: 0031 10 703 03 31
Postal address: Doctor Molewaterplein 40, 3015 GD, Rotterdam, The Netherlands
ORCID iD: 0000-0003-1829-9963

Intensified surveillance interview structure

Participants were interviewed in Dutch, their native language.

First, I want to ask you several questions regarding the surveillance program in general. Every year you undergo investigations to check the pancreas. You are a candidate for these check-ups because pancreatic cancer has occurred in your family and/or because a genetic mutation was found.

1. **What motivates you to participate in the surveillance program every year?**
   Open question, answers labelled as follows (multiple answers possible):

   a) Early detection of abnormalities
   b) Fear reduction
   c) For the benefit of my (younger) family members
   d) Because the doctor told me to
   e) For the benefit of scientific research
   f) It is the only thing that I can do myself
   g) Other, namely…

2. **What do you think of the initiative of a surveillance program?**
   Open question, answers labelled as follows:

   a) Very positive
   b) Somewhat positive
   c) Neutral
   d) Somewhat negative

3. **What do you think of the execution of the surveillance program?**
   Open question, answers labelled as follows:

   a) Good
   b) There is room for improvement
   c) Other, namely…

4. **In your opinion, what would be the ideal time between two check-ups?**
   Open question, answers labelled as follows:

   a) 3 months
   b) 6 months
   c) 12 months but shorter if abnormalities are found
   d) 12 months
   e) 24 months
   f) 60 months
   g) Other, namely…

5. **How long do you want to continue participating in a surveillance program?**
   Open question, answers labelled as follows:

   a) Forever, also beyond the maximum age set by the program
   b) Until the maximum age set by the program
   c) 10 years
   d) 5 years
   e) Shorter than 5 years, namely…
   f) I want to stop now

6. **To make images of the pancreas, they use an MR scan and/or an endoscopic ultrasound. With this last technique they insert a tube with a camera and echo into the mouth. How have you experienced the MR scan?**
   Open question, answers labelled as follows:

   a) Neutral, no problems at all
   b) Minor problems, for instance difficulties following breathing instructions, difficult to remain still for the entire investigation, problems with communication to the staff, slight feelings of claustrophobia
   c) Major problems, for instance severe claustrophobia, unable to complete the investigation
   d) I did not undergo an MR scan, because…

7. **How did you experience the endoscopic ultrasound?**
   Open question, answers labelled as follows:

   a) Neutral, no problems at all
   b) Minor problems, for instance some feelings of having insufficient sedation, slight anxiety for the endoscope
   c) Major problems, for instance always feeling as if sedation is insufficient, heavy anxiety for the endoscope
   d) I did not undergo an endoscopic ultrasound, because…

8. **Do you have a preference for one of the two tests?**
   Open question, answers labelled as follows:

   a) I have no preference
   b) I prefer EUS over MRI
   c) I prefer MRI over EUS
   d) I did not undergo both tests

9. **During the yearly check-ups, an abnormality was found in the pancreas, for which you have had extra check-ups, or are still undergoing extra check-ups. The next questions concern these extra check-ups. Did you experience these extra check-ups as different than the standard check-ups? If so, what was different?**
   Open question, answers labelled as follows (multiple answers possible):

   a) Similar
   b) More hassle or burdensome
   c) The investigations were different
   d) More anxiety or nervousness
   e) Other, namely…

10. **If the regular check-up frequency has been resumed, what was the reason for that?**
    Open question, answers labelled as follows:

    a) Abnormality remained stable, did not show growth
    b) Abnormality was deemed to be benign
    c) Abnormality was not found again
    d) I don't know
    e) Other, namely…

11. **How do you look back on this period of intensified surveillance?**
    Open question, answers labelled as follows:

    a) Mainly positive or relieved
    b) Neutral
    c) Negative but it was necessary
    d) Mainly negative

12. **Are you satisfied with the care you received in the hospital during this intensified surveillance period?**
    Open question, answers labelled as follows:

    a) Satisfied
    b) Neutral
    c) Not satisfied, because…

13. **Do you currently worry about the abnormality that was the reason for the intensified surveillance?**
    Open question, answers labelled as follows:

    a) Very
    b) Somewhat
    c) Neutral, not really
    d) Never
    e) There was no abnormality anymore

14. **Has your opinion of the surveillance program changed compared to when you first started participating? How has your opinion changed?**
    Open question, answers labelled as follows:

    a) It has become more positive
    b) It has remained unchanged
    c) It has become more negative
    d) Other, namely…

15. **We have come to the last part of the interview. In earlier research it was shown there are some factors that contribute to the amount of worries someone has about getting cancer. One of these factors is if someone has witnessed pancreatic cancer in the direct social circle, for instance in a family member. How worried are you about your family members getting pancreatic cancer?**
    Open question, answers labelled as follows:

    a) Very worried
    b) Not very worried
    c) Not worried at all

16. **Would you recommend your family members to also participate in a pancreatic cancer surveillance program, and why?**
    Open question, answers labelled as follows:

    a) Yes, I would recommend it, because…
    b) No, I would not recommend it, because…
    c) No, it is something they have to decide for themselves.
    d) I don't have family members that could participate/are allowed to participate

17. **Is there anything else you would like to say, or something we have forgotten to ask about but you feel is important to mention, regarding your experience with the surveillance?**

Surgery interview structure

Participants were interviewed in Dutch, their native language.

First, I want to ask you several questions regarding the surveillance program in general. Every year you undergo investigations to check the pancreas. You are a candidate for these check-ups because pancreatic cancer has occurred in your family and/or because a genetic mutation was found.

1. **What motivates you to participate in the surveillance program every year?**
   Open question, answers labelled as follows (multiple answers possible):

   a) Early detection of abnormalities
   b) Fear reduction
   c) For the benefit of my (younger) family members
   d) Because the doctor told me to
   e) For the benefit of scientific research
   f) It is the only thing that I can do myself
   g) Other, namely…

2. **What do you think of the initiative of a surveillance program?**
   Open question, answers labelled as follows:

   a) Very positive
   b) Somewhat positive
   c) Neutral
   d) Somewhat negative

3. **What do you think of the execution of the surveillance program?**
   Open question, answers labelled as follows:

   a) Good
   b) There is room for improvement
   c) Other, namely…

4. **In your opinion, what would be the ideal time between two check-ups?**
   Open question, answers labelled as follows:

   a) 3 months
   b) 6 months
   c) 12 months but shorter if abnormalities are found
   d) 12 months
   e) 24 months
   f) 60 months
   g) Other, namely…

5. **How long do you want to continue participating in a surveillance program?**
   Open question, answers labelled as follows:

   a) Forever, also beyond the maximum age set by the program
   b) Until the maximum age set by the program
   c) 10 years
   d) 5 years
   e) Shorter than 5 years, namely…
   f) I want to stop now

6. **To make images of the pancreas, they use an MR scan and/or an endoscopic ultrasound. With this last technique they insert a tube with a camera and echo into the mouth. How have you experienced the MR scan?**
   Open question, answers labelled as follows:

   a) Neutral, no problems at all
   b) Minor problems, for instance difficulties following breathing instructions, difficult to remain still for the entire investigation, problems with communication to the staff, slight feelings of claustrophobia
   c) Major problems, for instance severe claustrophobia, unable to complete the investigation
   d) I did not undergo an MR scan, because…

7. **How did you experience the endoscopic ultrasound?**
   Open question, answers labelled as follows:

   a) Neutral, no problems at all
   b) Minor problems, for instance some feelings of having insufficient sedation, slight anxiety for the endoscope
   c) Major problems, for instance always feeling as if sedation is insufficient, heavy anxiety for the endoscope
   d) I did not undergo an endoscopic ultrasound, because…

8. **Do you have a preference for one of the two tests?**
   Open question, answers labelled as follows:

   a) I have no preference
   b) I prefer EUS over MRI
   c) I prefer MRI over EUS
   d) I did not undergo both tests

9. **During the yearly check-ups, an abnormality was found in the pancreas, for which you eventually had surgery. The following questions are on your surgery and the period before and after that. Did you have additional check-ups before they decided to peform surgery?**
   Multiple choice question:

   a) Yes, I had additional check-ups
   b) No, they decided to perform surgery right when the abnormality was found

10. **If question 9 was "Yes": Did you experience these extra check-ups as different than the standard check-ups? If so, what was different?**
    Open question, answers labelled as follows (multiple answers possible):

    a) Similar
    b) More hassle or burdensome
    c) The investigations were different
    d) More anxiety or nervousness
    e) Other, namely…

11. **Were you scared for anything specific directly before the surgery?**
    Open question, answers labelled as follows (multiple answers possible):

    a) No specific fear
    b) For the surgery itself
    c) For the narcosis
    d) For the recovery and/or complications
    e) For the pathological outcome

12. **How do you look back to the surgery?**
    Open question, answers labelled as follows:

    a) As something positive
    b) Neutral
    c) As something necessary
    d) As something negative

13. **Are you satisfied with the care you received in the hospital in the period surrounding the surgery?**
    Open question, answers labelled as follows:

    a) Satisfied
    b) Neutral
    c) Dissatisfied

14. In your opinion, how was your recovery directly after your surgery?
Open question, answers labelled as follows:

a) Good, fast recovery and no complications
b) Fair, minor complications and/or longer recovery time than anticipated
c) Poor, major complications

15. How is your health at this moment?
Open question, answers labelled as follows:

a) Good, as before the surgery
b) Fair, improving, but not quite the same as before the surgery
c) Poor, because of the results of the surgery
d) Poor, because of reasons other than the surgery

16. Prior to surgery, it can never be said with 100% certainty what is the nature of the abnormality seen in the pancreas. Because of this, it can occur that after performing surgery, the abnormality turns out to be something benign, for which surgery may not have been required. How was this in your case? In your opinion, was it justified to have done the surgery?
Open question, answers labelled as follows:

a) Justified
b) Unjustified
c) I don't know

17. Do you know what was the result of the investigation of the part of the pancreas that was taken out by the surgery? What did they find?
Open question, answers labelled as follows:

a) Benign
b) Precursor lesion
c) It was unclear
d) Malignant
e) I don't know

18. According to your treating physician, was the surgery necessary/justified?
Open question, answers labelled as follows:

a) Yes, justified
b) No, unjustified
c) I don't know

19. Two of the tasks of the pancreas are to regulate the sugar levels in the blood and to create enzymes that help digest food, especially fats. After surgery of the pancreas, it is possible these tasks cannot be performed in the same way as before surgery. Diabetes and digestive complaints can therefore be consequences of surgery. Did you develop diabetes after the surgery? If so, are you treated with medication?
Open question, answers labelled as follows:

a) No
b) Yes, but it has been solved with diet changes
c) Yes, I am under treatment with tablets
d) Yes, I am under insulin treatment

20. Do or did you have digestive complaints, such as diarrhea, fatty and sticky feces, or abdominal pain? If so, are you or have you been treated with enzyme supplements?
Open question, answers labelled as follows:

a) No, no complaints
b) Yes, I had complaints, they have been solved with diet changes
c) Yes, I had complaints, they have been solved with diet changes and enzyme supplements
d) Yes, I had complaints, they have been solved with enzyme supplements
e) Yes, I have complaints, which could not be solved with diet changes and/or enzyme supplements

21. Do you experience pain as a result of the surgery?
Open question, answers labelled as follows:

a) No pain
b) Constant light pain
c) Some pain but decreasing over time
d) Moderate to severe pain

22. In hindsight, with all the knowledge you have now, would you again have chosen surgery in that same situation?
Open question, answers labelled as follows:

a) Yes, certainly
b) Yes, likely
c) Likely not
d) Certainly not

23. **Has your opinion of the surveillance program changed compared to when you first started participating? How has your opinion changed?**
    Open question, answers labelled as follows:

    a) It has become more positive
    b) It has remained unchanged
    c) It has become more negative
    d) Other, namely…

24. **We have come to the last part of the interview. In earlier research it was shown there are some factors that contribute to the amount of worries someone has about getting cancer. One of these factors is if someone has witnessed pancreatic cancer in the direct social circle, for instance in a family member. How worried are you about your family members getting pancreatic cancer?**
    Open question, answers labelled as follows:

    a) Very worried
    b) Not very worried
    c) Not worried at all

25. **Would you recommend your family members to also participate in a pancreatic cancer surveillance program, and why?**
    Open question, answers labelled as follows:

    a) Yes, I would recommend it, because…
    b) No, I would not recommend it, because…
    c) No, it is something they have to decide for themselves.
    d) I don't have family members that could participate/are allowed to participate

26. **Before I go on to the questions on quality of life, is there anything else you would like to say, or something we have forgotten to ask about but you feel is important to mention, regarding your experience with the surveillance?**

Now, I would like to continue with the last part of this interview. These are multiple choice questions and yes-or-no questions from a list that researchers use to investigate the quality of life. This means they contain questions regarding your mental and physical wellbeing. I will read the questions and answer options to you. Please try to answer the questions as accurately as you can.

1. **In general, would you say your health is…**

    a) Excellent
    b) Very good
    c) Good
    d) Fair
    e) Poor

2. **The next two questions concern activities you might do during a typical day. Please tell me if your health now limits you a lot, limits you a little, or does not limit you at all in these activities.**

    …Moderate activities, such as moving a table, pushing a vacuum cleaner, swimming or cycling.

    a) Yes, limited a lot
    b) Yes, limited a little
    c) No, not limited at all

3. **…Climbing several flights of stairs.**

    a) Yes, limited a lot
    b) Yes, limited a little
    c) No, not limited at all

4. **During the past 4 weeks, have you accomplished less than you would like as a result of your physical health?**

    a) Yes
    b) No

5. **During the past 4 weeks, were you limited in the kind of work or other regular daily activities you do as a result of your physical health?**
    a) Yes
    b) No

6. **During the past 4 weeks, have you accomplished less than you would like as a result of any emotional problems, such as feeling depressed or anxious?**

    a) Yes
    b) No

7. **During the past 4 weeks, did you not do work or other regular daily activities as carefully as usual as a result of any emotional problems, such as feeling depressed or anxious?**

    a) Yes
    b) No

8. **During the past 4 weeks, how much did pain interfere with your normal work, including both work outside the home and housework? Did it interfere…**

    a) Not at all
    b) A little bit
    c) Moderately
    d) Quite a bit
    e) Extremely

9. **During the past 4 weeks, how much of the time has your physical health or emotional problems interfered with your social activities like visiting with friends or relatives? Has it interfered…**

    a) All of the time
    b) Most of the time
    c) Some of the time
    d) A little of the time
    e) None of the time

The next questions are about how you feel and how things have been with you during the past 4 weeks. As I read each statement, please give me the answer that comes closest to the way you have been feeling; is it all of the time, most of the time, a good bit of the time, some of the time, a little of the time, or none of the time?

**How much of the time during the past 4 weeks…**	All	Most	A good bit	Some	A little	None
**10. …Have you felt calm and peaceful?**	1	2	3	4	5	6
**11. …Did you have a lot of energy?**	1	2	3	4	5	6
**12. …Have you felt downhearted and blue?**	1	2	3	4	5	6
